# Metagenomic Analysis of RNA Viruses in a Fresh Water Lake

**DOI:** 10.1371/journal.pone.0007264

**Published:** 2009-09-29

**Authors:** Appolinaire Djikeng, Ryan Kuzmickas, Norman G. Anderson, David J. Spiro

**Affiliations:** 1 Infectious Diseases Group, The J. Craig Venter Institute (JCVI), Rockville, Maryland, United States of America; 2 Viral Defense Foundation, Kensington, Maryland, United States of America; Georgia Institute of Technology, United States of America

## Abstract

Freshwater lakes and ponds present an ecological interface between humans and a variety of host organisms. They are a habitat for the larval stage of many insects and may serve as a medium for intraspecies and interspecies transmission of viruses such as avian influenza A virus. Furthermore, freshwater bodies are already known repositories for disease-causing viruses such as Norwalk Virus, Coxsackievirus, Echovirus, and Adenovirus. While RNA virus populations have been studied in marine environments, to this date there has been very limited analysis of the viral community in freshwater. Here we present a survey of RNA viruses in Lake Needwood, a freshwater lake in Maryland, USA. Our results indicate that just as in studies of other aquatic environments, the majority of nucleic acid sequences recovered did not show any significant similarity to known sequences. The remaining sequences are mainly from viral types with significant similarity to approximately 30 viral families. We speculate that these novel viruses may infect a variety of hosts including plants, insects, fish, domestic animals and humans. Among these viruses we have discovered a previously unknown dsRNA virus closely related to Banna Virus which is responsible for a febrile illness and is endemic to Southeast Asia. Moreover we found multiple viral sequences distantly related to Israeli Acute Paralysis virus which has been implicated in honeybee colony collapse disorder. Our data suggests that due to their direct contact with humans, domestic and wild animals, freshwater ecosystems might serve as repositories of a wide range of viruses (both pathogenic and non-pathogenic) and possibly be involved in the spread of emerging and pandemic diseases.

## Introduction

In recent years viral metagenomics has opened a window on the complexity of several ecosystems including marine water, marine sediment, animal feces, human feces and nasal swabs [1]–[4] and more recently it has even been used to explore the Yellowstone Hot Springs [5]. Most of these studies have not only uncovered new viruses but have also increased our knowledge of viral diversity and ecology. It is now well established – contrary to the previous assumption that only dsDNA viruses were present in marine environments [6]–[8] – that both RNA (ss and ds) and DNA (ss and ds) viruses are present in marine environments, the ocean and probably many other environments including freshwater lakes (this study). In addition to a simple description of the presence of known and unknown viruses in these environments there have also been some attempts to 1) understand the biological relevance of these findings and 2) estimate the representation of the overall viral biomass. Rohwer and colleagues estimated that approximately 5,000 viral genotypes could be present in approximately 200 liters of sea water [9]. In fact from this study, about 75% of these sequences do not match any previously described sequences in the public domain databases. Angly and colleagues demonstrated a global viral diversity consisting of greater than one hundred thousand viruses as well as localized regional diversity [10]. The vast majority of sequences found in these studies belong to novel subgroups that are not represented by cultured isolates. All of the conserved gene studies suggest that environmental viral diversity is extremely rich and essentially uncharacterized. Nevertheless, almost identical sequences of some DNA viruses such as bacteriophages were found at a number of different locations and environments across the world [11], leading to a model where viruses demonstrate high local diversity but relatively lower global genetic diversity probably due to horizontal gene transfer [9], [12]. The study on Yellowstone Hot Springs focusing on DNA viruses further demonstrates that viruses are also found in geothermal environments probably in close association with their hosts [5].

Given the diversity of RNA viruses in marine communities and the importance of RNA viruses as human pathogens and emerging disease threats, we surveyed the freshwater body Lake Needwood focusing on RNA viruses. Lake Needwood provides a snapshot of a body of water with surrounding varying land use distribution with urban (52%), agricultural (22%) and forest (25%) in its drainage basin. We collected lake water samples in June 2006 and November 2007. Each sample was processed for enrichment of viral particles, construction of libraries of DNA amplicons, sequencing and data analysis. To the best of our knowledge this is the first comprehensive metagenomic study of RNA viruses in a freshwater lake.

## Results

### 1) Identification of RNA viruses in freshwater lake

We conducted two seasonal samplings of Lake Needwood with an emphasis on investigating the diversity and the ecology of RNA viruses. Lake Needwood is located in Montgomery County, Maryland, USA. During each of the two sampling expeditions, we collected approximately 70 liters of water from the surface of the Lake about five meters away from the shore. The expeditions and collections were conducted in November 2006 and in June 2007. From each sample, viral particles were concentrated using the tangential flow filtration [13]. Concentrated viral particles were treated with nucleases (DNase I and RNase A) to eliminate free nucleic acids from degraded cells. Nuclease-treated samples were processed for total viral RNA extraction. Extracted viral RNA was amplified using the random priming mediated sequence independent single primer amplification (RP-SISPA) as previously described [14]. Each amplified sample yielded a library of amplicons containing DNA fragments of sizes ranging from ∼300 to ∼2600 kb. In the first instance, for each sample, a library of random plasmid clones was constructed by cloning of amplicons (of 500–1000 nucleotides in size) into the Topo TA vector. Random recombinant plasmid clones were first selected from each library for Sanger sequencing. A total of 4156 Sanger sequencing reads were generated from the library constructed using viral RNA prepared from enriched viral particles of the November 2006 sample. A total of 3928 Sanger sequencing reads were generated from the library constructed using viral RNA prepared from enriched viral particles of the June 2007 sample. Preliminary analysis of the Sanger sequencing data revealed a great diversity of sequences found based on their BLASTX matches. To better explore the viral diversity through greater sequence coverage, we used the 454 pyrosequencing method to generate many more sequences [15], [16]. For the 454 pyrosequencing, DNA fragments ranging from 300 to 500 nt were size-selected from the library of DNA fragments generated by the RP-SISPA. The size selected DNA samples were used for 454 pyrosequencing. A total of 219402 and 283973 reads with an average read length of 230 nt were generated using the 454 FLX genome sequencer (454 Life Sciences, CT) from November 2006 and June 2007 samples, respectively. Preliminary analysis of the two data subsets (Sanger and 454) revealed very similar profiles in terms of redundancy and diversity. Specifically, MEGAN analysis [17] of each of the two data sets revealed very similar taxonomic diversity profiles (data not shown).

**Figure 1:**
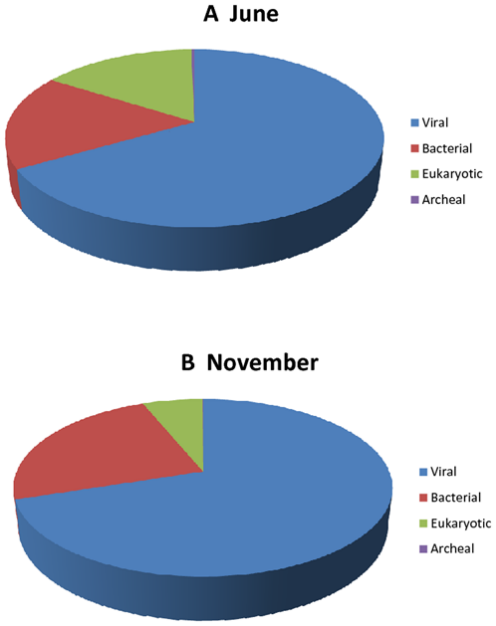
Taxonomic classification of assemblies. Assemblies were classified based on comparison to the CAMERA database using the BLASTX algorithm and an e-value of 1×10e-5 or lower. Sequences in assemblies without significant matches to existing protein sequences (e-value>1E-5) were classified as “Unknown”. The remaining sequences were classified based on best BLASTX hits for their assemblies. Of the “known” sequences, 67% of the November sample and 70% of the June sample had homology to published viral sequences.

Table 1 provides a combined summary analysis of sequence reads generated by Sanger and 454 pyrosequencing. All reads were trimmed to remove primer sequences and low-quality sequences. Hybrid assemblies combining Sanger and 454 reads were generated using the Newbler Assembler version 1.1.03.19 with a 50 bp minimum match length and 90% identity threshold as previously described [18]. A total of 15,376 and 10,133 contiguous assemblies ranging in size from 94 bp to 5719 bp were generated from November and June, respectively (Table 1). Assemblies were categorized using BLASTX homology search against the CAMERA non-redundant amino acid database [19]. To simplify our categorizations, assemblies matching sequences in the database with an e-value of 10e-5 or lower were assigned the identity of the top BLASTX match. All subsequent phylogenetic classification and analysis were performed based on the BLASTX results. Our analysis showed that a large majority (∼66%) of these assemblies had no significant homology to any known sequences of viral, bacterial, eukaryotic and archaeal origin (Fig. 1A and 1B). The remaining reads (∼34%) could be broadly classified as viral, bacterial, archaeal and eukaryotic origin. For the November 2006 sample, matching reads were distributed as follow: 66.7% (viral origin), 17.4% (bacterial origin), 0.3% (archaeal origin) and 15.7% (eukaryotic origin). For the June 2007 sample, matching reads were distributed as follow: 69.9% (viral origin), 23.8% (bacterial origin), 0.05% (archaeal origin) and 6.1% (eukaryotic origin). As expected, the majority of reads with significant homology to known sequences were of viral origin. Although a significant portion of sequences in each sample has been classified as eukaryotic or bacterial in origin, subsequent analysis showed that 825 November and 2352 June “bacterial” sequences match putative prophage elements in sequenced bacterial genomes, suggesting that some portions of these sequences may still be of viral origin. Furthermore, BLASTN analysis showed that only 1% of reads for November and 2% of reads for June were homologous to any ribosomal RNAs. This ribosomal contamination was expected but appeared to be lower than that found in previous viral metagenomic studies [2], [3], [8] and provides further validation that sequences from cellular organisms were largely removed by our protocol.

**Table 1:** Total sequences metrics and preliminary classification.

	NOVEMBER	JUNE
	Raw	Percent	Raw	Percent
454 Reads	219402		283973	
Sanger Reads	4156		3928	
Total reads in assemblies	163507	73.10%	165720	57.60%
Total Contigs	15376		10133	

**Figure 2:**
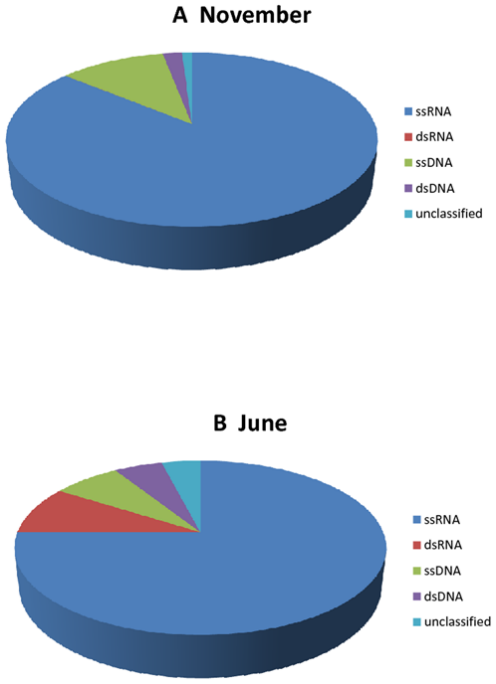
Composition of viral types. Assemblies were assigned into one of five categories based on nearest BLASTX homology results. For both the November and June samples, approximately 87% of all viral sequence reads for each season were in assemblies matching RNA viruses.

Further classification based on viral group showed that nearly 90% of all viral sequences are homologous to RNA viruses, validating the utility of this protocol for targeting RNA viruses (Fig. 2A and 2B). Based on BLASTX results, we classified sequences with homology to known viruses into ssRNA (single stranded), dsRNA (double stranded), ssDNA and dsDNA viruses. The majority of sequences with viral homology (86% for November and 75% for June) matched ssRNA viruses. Viruses of the dsRNA group were far (8.6% versus 0.01%) more abundant in the June sample as compared to the November sample. The next large group of reads matched (10.6% in the November sample and 6.6% in the June sample) ssDNA viruses. Double stranded DNA viruses were found at 2.3% and 5.5% in the November and June samples, respectively. Most of these dsDNA viruses represented phages known to infect bacteria.

### 2) Diversity of RNA viruses in fresh lake water

**Figure 3:**
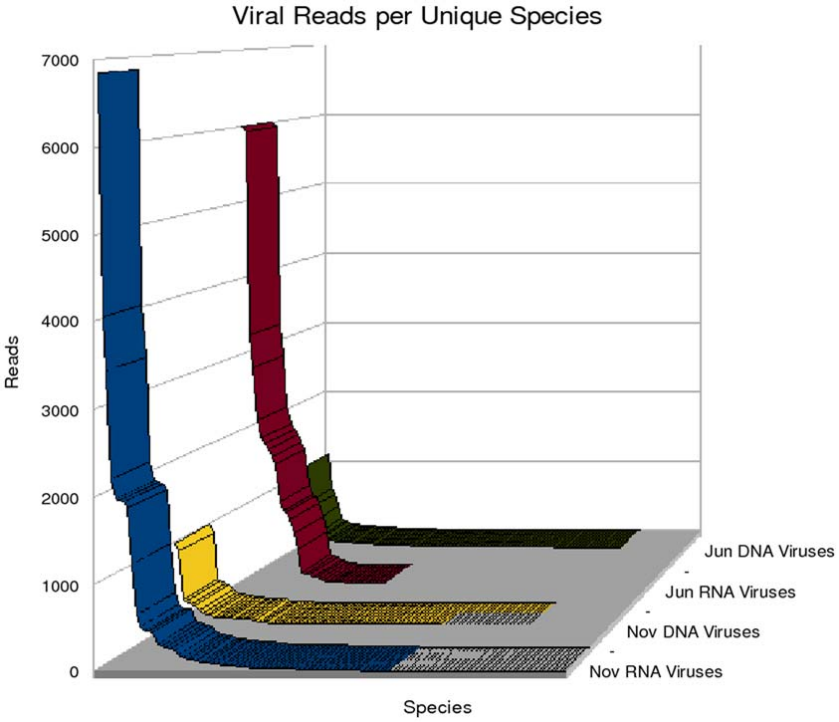
Distribution of viral reads by viral types. Names were assigned to assemblies based on the best BLASTX match. Assemblies with the same virus name were grouped together, and the numbers of reads comprising these assemblies were added to generate a reads per unique types value. Although RNA viruses were the target of this study and the best-represented in the data, DNA viruses have been included as well for comparison.

To examine the diversity of viruses present in Lake Needwood, assemblies were assigned to viral families based on the taxonomy of their closest relatives as judged by BLASTX analysis. Over 25 viral families were detected with the majority of the assemblies homologous to ssRNA viruses. These assemblies were classified into DNA and RNA viruses (Figure 3). The total number of hits to DNA viruses remained constant in both June and November samples. In contrast, we observed a slight shift in RNA viruses with more homologous sequences in November as compared to June. The relatively high number of RNA viruses in the November sample could also be correlated with a broader classification of viral families observed and reported in Tables 2 and 3.

**Table 2:** RNA Viruses Families in Lake Needwood.

Family	Host	Season
Marnaviridae	Heterokonts	Both
Unclass. ssRNA	Heterokonts	Both
Unclass. ssRNA	Plants	Both
Unclass. ssRNA	Mollusks	Both
Nodaviridae	Fish	Both
Dicistroviridae	Arthropods	Both
Unclass. Umbravirus	Plants	Both
Unclass. Sobemovirus	Plants	Both
Tombusviridae	Plants	Both
Comoviridae	Plants	Both
Picornaviridae	Birds/Mammals	June
Reoviridae	Arthropods	June
Unclass. Picobirnavirus	Mammals	June
Partitiviridae	Plants	June
Leviviridae	Bacteria	November
Hepeviridae	Birds/Mammals	November
Orthomyxoviridae	Birds/Mammals	November
Flaviviridae	Mammals	November
Iflaviridae	Arthropods	November
Bromoviridae	Plants	November
Luteoviridae	Plants	November
Flexiviridae	Plants	November
Tetraviridae	Plants	November
Unclass. Tobamovirus	Plants	November
Tymoviridae	Plants	November
Sequiviridae	Plants	November
Unclass. Iflavirus	Arthropods	November
Unclass. Nora virus	Arthropods	November

Classified RNA viral families are listed along with seasonal and host distribution. Families were assigned to sequences based on best BLASTX matches (e-value<1e-5 or lower). Host type was assigned using viral species (not viral family) data, and is therefore not reflective of the full host range for a given viral family.

**Table 3:** Most abundant RNA viruses detected in Lake Needwood.

June			November		
Species	Number of sequences	Largest contig	virus	Number of sequences	Largest contig
Acute bee paralysis virus	9	377	Acheta domesticus virus	25	636
Angelonia flower break virus	2	234	Acute bee paralysis virus	38	901
Aphid lethal paralysis virus	72	797	Angelonia flower break virus	5	241
Artichoke mottled crinkle virus	2	215	Aphid lethal paralysis virus	463	1412
Avian encephalomyelitis virus	1559	1271	Artichoke mottled crinkle virus	2	234
Banna Virus	3261	2402	Atlantic cod nervous necrosis virus	2	241
Black queen cell virus	7	340	Atlantic halibut nodavirus	6	813
Cowpea mosaic virus	1912	852	Avian hepatitis E virus	24	1180
Cricket paralysis virus	1071	442	Banna virus	3	241
Cucumber necrosis virus	8	329	Bean leafroll virus	2	135
Drosophila C virus	5996	4475	Bean pod mottle virus	151	2642
Galinsoga mosaic virus	2826	394	Beet black scorch virus	248	578
Grapevine Algerian latent virus	4	338	Blackberry virus Z	18	830
Groundnut rosette virus	3	236	Boolarra virus	4	164
Havel river tombusvirus	4	151	Bovine kobuvirus	5	229
Heterosigma akashiwo RNA virus SOG263	125	1874	Broad bean wilt virus 1	15	955
Himetobi P virus	2140	3119	Carnation Italian ringspot virus	25	450
Homalodisca coagulata virus-1	74	975	Carnation mottle virus	93	899
Human enterovirus 94	52	1269	Carnation ringspot virus	2	178
Human picobirnavirus	115	1174	Cocksfoot mottle virus	47	645
Israel acute paralysis virus of bees	940	2228	Cowpea mosaic virus	440	3645
Maize chlorotic mottle virus	833	261	Cowpea mottle virus	13	349
Oat chlorotic stunt virus	3	166	Cricket paralysis virus	4060	2803
Olive latent virus 1	36	1111	Cucumber Bulgarian latent virus	35	481
Pariacato virus	446	764	Cucumber leaf spot virus	79	1164
Pea stem necrosis virus	4	240	Cucumber necrosis virus	41	718
Pear latent virus	9	288	Cymbidium ringspot virus	5	238
Pelargonium line pattern virus	2	483	Dendrolimus punctatus tetravirus	51	843
Pieris rapae virus	1762	1570	Dicentrarchus labrax nervous necrosis virus	5	730
Rhizosolenia setigera RNA virus	5937	2805	Drosophila C virus	3447	1765
Rhopalosiphum padi virus	955	1839	Echovirus 23 strain Williamson	6	402
Ryegrass mottle virus	2	241	Enterobacterio phage MS2	3	241
Schizochytrium single-stranded RNA virus	661	2036	Euprosterna elaeasa virus	2	224
Sclerophthora macrospora virus A	11	337	Flock house virus	2	229
Sclerophthora macrospora virus B	145	1109	Fragaria chiloensis latent virus	5	583
Solenopsis invicta virus 1	24	689	Galinsoga mosaic virus	187	1941
Tobacco necrosis virus D	1	128	Grapevine Algerian latent virus	9	178
Tomato bushy stunt virus	3	170	Helicoverpa armigera stunt virus	3	219
Triatoma virus	1829	590	Hepatitis E virus	133	3258
unidentified chinese clam virus 16–50	1660	272	Heterosigma akashiwo RNA virus SOG263	20	383
Vicia cryptic virus	9	217	Hibiscus chlorotic ringspot virus	1984	4124
			Himetobi P virus	76	883
			Homalodisca coagulata virus-1	263	1606
			Infectious flacherie virus	462	4424
			Influenza A virus (AternAustralia13632004 (H2N5)	4	230
			Israel acute paralysis virus of bees	2202	1982
			Japanese iris necrotic ring virus	40	952
			Kashmir bee virus	655	4407
			Leek white stripe virus	165	1837
			Lisianthus necrosis virus	37	624
			Maize chlorotic dwarf virus	28	546
			Maize chlorotic mottle virus	1874	1074
			Maize necrotic streak virus	2	134
			Maize white line mosaic satellite virus	74	916
			Maize white line mosaic virus	2	177
			Melon necrotic spot virus	18	401
			Nodamura virus	5	354
			Nootka lupine vein-clearing virus	6	241
			Nora virus	6	193
			Oat chlorotic stunt virus	513	703
			Odontoglossum ringspot virus	95	1907
			Olive latent virus 1	1942	1530
			Panicum mosaic virus	4	151
			Pariacato virus	35	723
			Pea enation mosaic virus-2	33	695
			Pea stem necrosis virus	74	1109
			Pear latent virus	18	397
			Pelargonium chlorotic ring pattern virus	2	241
			Pelargonium line pattern virus	57	1058
			Pelargonium necrotic spot virus	25	863
			Perina nuda virus	3	104
			Physalis mottle virus	7	765
			Pieris rapae virus	8	466
			Plautia stali intestine virus	28	441
			Pothos latent virus	69	925
			Redspotted grouper nervous necrosis virus	4	709
			Rhizosolenia setigera RNA virus	6856	3726
			Rhopalosiphum padi virus	12	402
			Ribgrass mosaic virus	4	240
			Rice tungro spherical virus	28	1179
			Rice yellow mottle virus	3	687
			Ryegrass mottle virus	275	2497
			Sacbrood virus	3	229
			Saguaro cactus virus	4	339
			Schizochytrium single-stranded RNA virus	34	1161
			Sclerophthora macrospora virus A	1238	3563
			Sclerophthora macrospora virus B	1960	2335
			Sesame necrotic mosaic virus	102	1049
			Solenopsis invicta virus 1	26	650
			Solenopsis invicta virus 2	5	397
			Southern bean mosaic virus	3	217
			Sowbane mosaic virus	5	720
			Soybean dwarf virus	5	124
			Striped Jack nervous necrosis virus	3	634
			Taura syndrome virus	147	1929
			Tobacco bushy top virus	16	700
			Tobacco mild green mosaic virus	21	583
			Tobacco mosaic virus	13	422
			Tobacco mottle virus	5	227
			Tobacco necrosis satellite virus	43	874
			Tobacco necrosis virus A	313	1764
			Tobacco necrosis virus D	39	720
			Tomato bushy stunt virus	15	711
			Tomato white ringspot virus	5	241
			Triatoma virus	108	1028
			Tulip virus X	2	242
			Turnip vein-clearing virus	103	1594
			unidentified chinese clam virus 16–50	291	332

**Figure 4:**
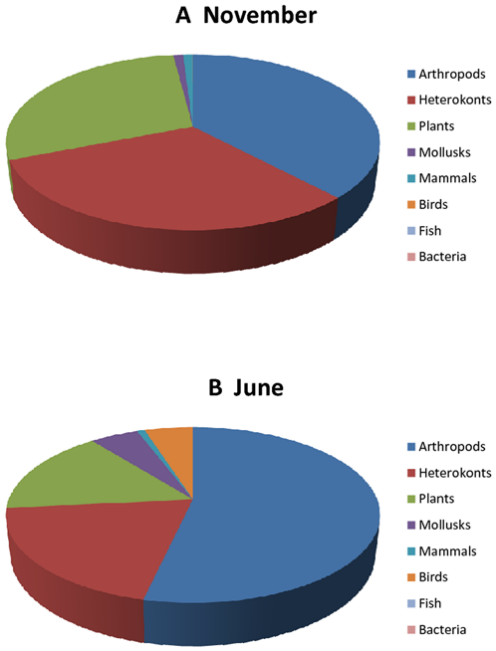
Distribution of potential hosts of RNA viruses. BLASTX results were used to classify viral hits. The information obtained from the bibliographic description of the identified virus was then used to identify the most probably host.

The overall taxonomic distribution of RNA viruses detected in Lake Needwood showed a marked change in viral diversity from June to November with overlapping but largely unique populations of viral families and types in each of the seasons monitored. Analysis of best BLASTX hits to assemblies showed that the viral profile is representative of many viruses known to infect many species of protists, plants, and animals associated with freshwater environments (Figure 4). Unexpectedly, the virus host population did not appear to be dominated by unicellular organisms as would be predicted by the biomass, since the majority of assemblies detected were homologous to viruses which infect higher and complex organisms such as plants, crustaceans, insects, fishes, and mammals. Of significance – in terms of public health, agriculture and land development – we detected a number of assemblies homologous to viruses reported to be causative agents of diseases of plants, insects, livestock, aquaculture and humans (Table 2).

While the two data sets show broad taxonomic similarity, there is however a notable seasonal variation at more specific levels. Nucleotide sequence analysis of representative sequences of families reported in Tables 2 and 3 showed that even with a fairly permissive classification, there is little identical sequence overlap between the two seasons. It should be noted however, that this analysis is coverage-limited; hence a given virus could be present in both seasons yet have sequence coverage on separate parts of the genome for each season and therefore not be included in this analysis. To appreciate the variation of RNA viruses between the samples, the BLASTX hits were classified by viral family and by host type. Table 2 also shows a breakdown of viruses by families. While overlap exists between the two samples, the majority of viral families identified are unique to one season. Classification of RNA virus reads by host type also shows noticeable seasonal variation. Figure 4 shows that the November sample is dominated by RNA viruses infecting plants and heterokonts (diatoms, algae) comprising 60% of the sample, and viruses infecting animals comprising under 40% of the sample. The June sample inverts these results with RNA viruses that infect animals making up about 64% of the sample, and viruses infecting plants and heterokonts now only representing about 36% of the total.

To begin to gain insights into the relative abundance of the viruses detected during the two time points, we sought to determine the relative abundance of the most numerous viruses by grouping sequencing reads. Table 3 provides the breakdown of the number of reads assigned to each virus detected. Once again this distribution confirmed a much greater diversity in the November data set as compared to the June data set. Two viruses showed clear demarcation of detection during one season and very low or none during the other season. The influenza A virus was detected only in the November data set. Similarly, the banna virus – so far reported only in tropical climates – and the avian encephalomyelitis virus were largely detected in the June data set. In addition based on the number of reads assembled and the length of the largest contigs, viruses such as the banna virus, himetobi virus, the Israel acute paralysis virus, the rhopalosiphum padi virus among others appear to be predominantly found in the June data set. The cricket paralysis virus appears to be predominantly found in the November data set. In contrast some viral types, such as the drosophila C virus, the rhizosolenia setigera RNA virus, were present in both data sets. We observed a significant increase of plant viruses in November as compared to June. This may correspond to the presence of many plants infected with viruses in the Lake environment during the fall season. The same trend may be true for animal viruses. However details studies remain important to effectively correlate the abundance of viruses and their hosts during different seasons.

### 3) Comparison of Lake Needwood metagenomics data sets to other metagenomics data sets

We further evaluated the taxonomic diversity of the Lake Needwood data set by comparing it to available sequence data sets generated from other viral metagenomics studies available on the CAMERA website (http://camera.calit2.net). These studies included the global ocean survey (GOS; [20], [21]), marine viromes [10], acid mine [22], farm soil and whale fall [23], the Chesapeake Bay Virioplankton [24], the sludge communities [25], and the deep Mediterranean plankton [26]. Table 4 summarizes the BLAST comparison of the lake Needwood data sets with other selected metagenomics data sets. The GOS data set, which is currently by far the largest and most complex, contained sequences which were homologous with between 15.5% (for June 2007) and 18% (for November 2006) of the Lake Needwood data set. Other metagenomics datasets contained sequences similar to between 0.5 and 8% of our data. This relatively small overlap suggests in part that each environment may harbor a core community of microorganisms which may significantly differ from one ecosystem to another. On the other hand, the little overlap observed between our data and existing metagenomics data sets can also be explained by the fact that our studies contrary to other focused mainly on the identification of RNA viruses. Overall, we observed similar overlapping profiles for both the June and November samples indicating that although there may be seasonal changes in the same environment the overall composition in the viral community and other microorganisms remains tightly comparable.

**Table 4:** Comparison with other viral metagenomics data sets.

Metagenomic Dataset	November		June	
	Hits	Percent Reads	Hits	Percent Reads
Coastal RNA Virus Communities	396	17.4% (28376)	140	13.3% (22092)
GOS	3512	17.8% (29078)	2014	15.5% (25683)
Marine Viromes	1337	8.3% (13537)	980	6.3% (10502)
Acid Mine	234	1.9% (3093)	152	3.3% (5427)
Farm Soil	81	0.5% (855)	55	1.8% (3060)
Whale Fall	63	0.3% (568)	30	1.3% (2222)
Deep Mediterranean plankton	33	0.1% (188)	36	1% (1685)
Sludge communities	628	4.7% (7649)	42	1.7% (2872)
Chesapeake Bay Virioplankton	43	0.4% (584)	53	0.9% (1572)

### 4) Viruses with potential impact on agriculture and public health

**Figure 5:**
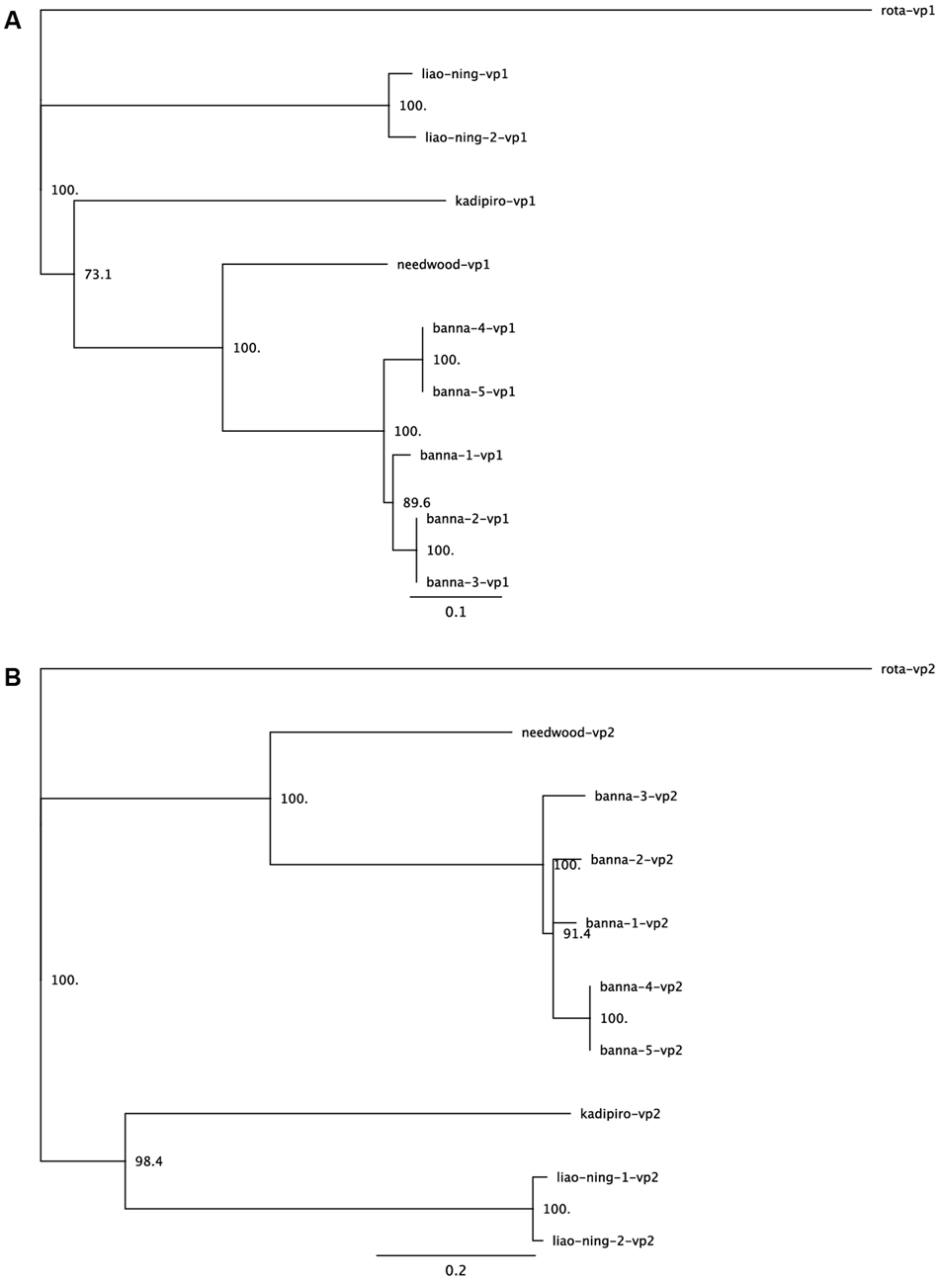
Phylogenetic trees of Banna virus. The entire vp1 and vp2 segments were chosen for sequence comparison analysis. Other sequences used for the analysis were downloaded from the NCBI database. All sequences were analyzed using ClustalX with default parameter settings as described in Materials and Methods. Consensus tree bootstrapping was performed with Geneious 4.0.4 using the neighbor-joining method and 1,000 samples.

The level of complexity and diversity of viral hits in our data sets was also underscored by the significantly high number of assemblies bearing homology with well known viruses. Some of these known viruses, on the basis of their putative hosts, could potentially affect both human and animal health directly or indirectly (Table 5). Of interest, we have identified two candidate viruses with significant sequence similarity to the Banna virus and the Israel acute paralysis virus of bees. In particular, we found eight assemblies ranging in size from 240–2400 nt and represented by a total of 3261 sequences from the June sample and 3 sequences from the November sample covering larges segments of 9 out of 12 genome segments and comprising more than 60% of the total genome of the Banna virus. Banna virus is a segmented dsRNA virus and one of the 3 members of the Seadornaviridae genus. It is the causative agent of a febrile illness and to date has been reported only in eastern and southeastern Asia. It has been classified as a biosafety level three arboviral agent [27]. It is further known as a mosquito-borne virus, endemic to eastern and southeastern Asia and has been reported to infect humans, leading to encephalitis and flu-like symptoms [28]. Prevalence of its infection is unclear due to symptomatic similarity with other causes of encephalitis. The homology between the novel Lake Needwood Seadornavirus virus and Banna virus appears to be closer than that of Banna with the Kadipiro virus, a previously reported member of the Seadornavirus genus (Table 6). We were able to assemble 80% of the amino acid sequence for the VP1 segment (RNA-dependent RNA polymerase of Needwood virus). We also generated a complete sequence of the VP2 segment (the inner-layer coat protein). To further determine the relatedness of the Banna virus detected in North America to the ones reported elsewhere, we conducted a phylogenetic analysis using sequences from both VP1 and VP2 segments (Figure 5A and 5B). The phylogenetic data suggests the Needwood Banna virus does not group with other viruses. This may be an indication of the difference between the strains from the two geographically distinct environments. There may have been a speciation event creating Banna Virus and the Needwood Banna-like Virus which occurred after the event that separated Banna Virus from Kadipiro and Liao Ning viruses (Figure 5A and 5B).

**Table 5:** Summary of candidates disease causing and novel viruses identified in Lake Needwood.

Viruses	November (e-value)	November (potential host)	June (e-value)	June (potential host)
Ryegrass mottle virus	0	Plant		
Infectious flacherie virus	1.55e-154	Insect-Silkworm		
Hepatitis E	1.44e-149	Human		
Cucumber necrosis virus	6.26e-115	Plant		
Cocksfoot mottle virus	3.32e-112	Plant		
Drosophila C virus	2.43e-092	Insect		
Sclerophthora macrospora virus A	5.89e-082	Marine Fungus		
Aphid lethal paralysis virus	1.20e-078	Insect		
Rhizosolenia setigera RNA virus	1.10e-077	Diatom		
Homalodisca coagulata virus1	1.99e-077	Insect		
Atlantic halibut nodavirus	5.39e-036	Fish		
Taura syndrome virus	2.19e-041	Shrimp		
Kashmir bee virus	2.20e-024	Insect		
Redspotted grouper nervous necrosis virus	3.40e-029	Fish		
Banna virus			0	Human-BSL3 arbovirus
Drosophila C virus			1.04e-093	Insect
Olive latent virus 1			1.53e-091	Plant
Rhizosolenia setigera RNA virus			1.64e-068	Diatom
Israel acute paralysis virus of bees			2.00e-58	Insect-Colony Collapse Disorder
Schizochytrium singlestranded RNA virus			1.91e-039	Marine fungoid protist
Heterosigma akashiwo RNA virus			3.86e-027	Diatom
Solenopsis invicta virus 1			3.29e-026	Insect-Red Fire Ants
Subterranean clover stunt virus			6.68e-026	Peas, beans and clover
Aphid lethal paralysis virus			7.54e-026	Insect
Himetobi P virus			8.56e-024	Insect
Homalodisca coagulata virus1			2.34e-020	Insect

**Table 6:** Summary coverage of a Banna-like virus.

Lake Needwood banna-like virus			Kadipiro virus	
Segment	Coverage	Identity	Segment	Identity
VP1	83%	63%	VP1	41%
VP2	100%	46%	VP2	27%
VP3	58%	55%	VP3	36%
VP4	9%	56%	VP4	32%
VP5	83%	46%	VP5	26%
VP6	57%	45%	VP6	26%
VP7	0%	n/a	VP7	28%
VP8	100%	41%	VP8	23%
VP9	100%	35%	VP9	n/a
VP10	0%	n/a	VP10	24%
VP11	47%	66%	VP11	37%
VP12	n/a	n/a	VP12	n/a

**Figure 6:**
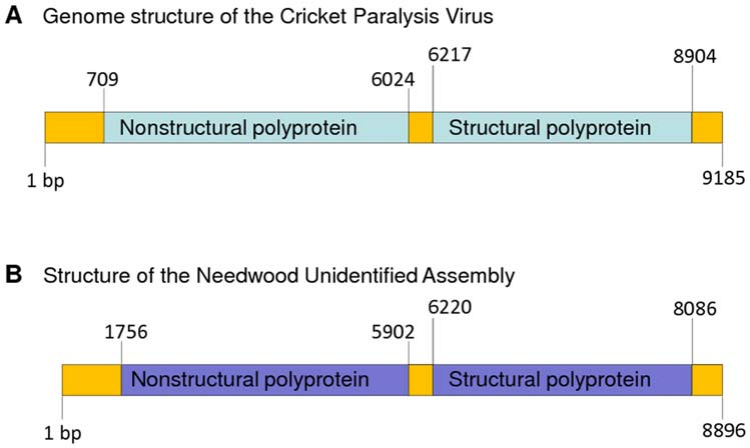
Genomic organization of a novel paralysis virus. (A) Genomic organization of cricket paralysis virus. (B) Genomic organization of the putative novel paralysis virus identified in Lake Needwood. Two contiguous sequences with sizes of 6000 and 1500 nucleotides assembled from combined June and November reads exhibited significant homology with the cricket paralysis virus. Using targeting amplification a DNA fragment of 700 nt was amplified, sequenced and used to link the two contigs thus generating a consensus sequence of 8086 nt. Using BLASTX we mapped the boundaries of the two (non structural and structural) polyproteins. (C) Phylogenetic analysis of the Lake Needwood virus assembly with homologous paralysis viruses. A region (containing ∼600 amino acids residues) of the replicase polyprotein was used for phylogenetic analysis after multiple sequences alignment using ClustalX with default parameter settings as described in Materials and Methods. Consensus tree bootstrapping was performed with Geneious 4.0.4 using the neighbor-joining method and 1,000 samples.

Additionally we have detected several distinct viruses with significant homology to known insect paralysis viruses from the ssRNA family Dicistroviridae [29], [30]. A nearly complete assembly of a novel insect paralysis virus was generated and appeared to share homology and synteny with Israeli Acute Paralysis virus, Acute Bee paralysis virus, Cricket paralysis virus, and Kashmir bee virus (Figure 6). We used a ∼600 amino avid sequence of the replicase polyprotein to perform a phylogenetic analysis. Our data indicate that the unknown virus assembly identified in Lake Needwood appear to be distant from the Israel acute paralysis virus, the acute bee paralysis virus and the Kashmir bee virus. This may be indicative of the presence of a putative new paralysis virus closely related to previously describe homologous viruses. Further studies specifically focusing on full genome sequencing, comparative genomes and host identification are required to conclude on the identification of a new paralysis virus.

### 5) Diversity of circoviruses

**Figure 7:**
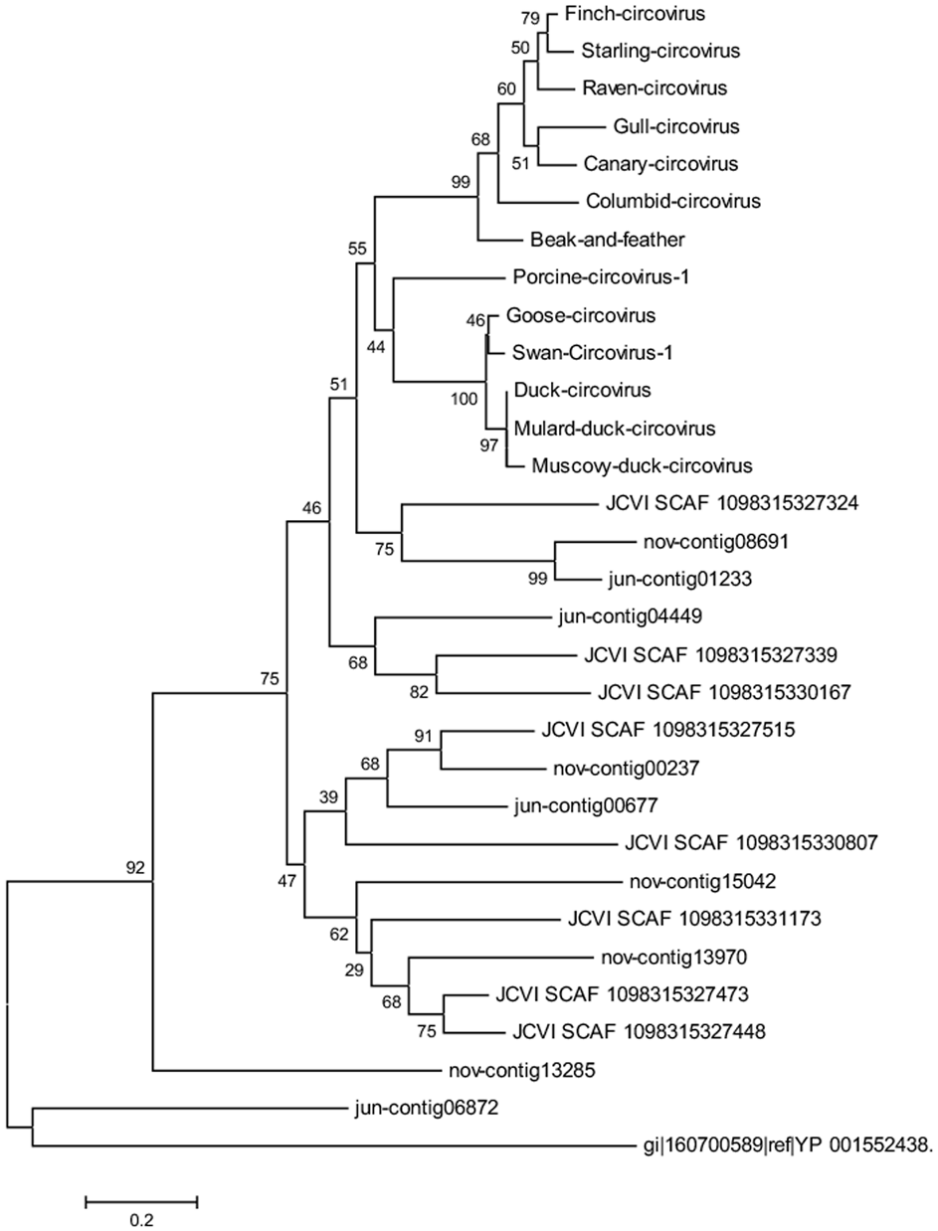
Phylogenetic tree of circoviruses. A region of 90 amino at the 3′ end of the circoviruses polymerase gene was selected for sequence comparison analysis. Other sequences were downloaded from the GOS and the NCBI databases. Selected sequences were analyzed using ClustalX with default parameter settings as described in Materials and Methods. Consensus tree bootstrapping was performed with Geneious 4.0.4 using the neighbor-joining method and 1,000 samples.

Despite experimental procedures that we establish to enrich for RNA viruses, a significant number of sequence reads mapped to DNA viruses both ssDNA and dsDNA. While the majority of dsDNA viruses were bacteriophages, the ssDNA viruses contained numerous sequences with significant homology to circoviruses. Circoviruses represent a family of small, icosahedral and non-enveloped viruses with circular genome composed of ssDNA. Several members of this family have been identified and associated with diseases in birds and mammals [31], [32]. Based on recent data, they form the circoviridae family which comprises three types and two genera including TTV viruses and TT-like mini virus. Their genome is made up of a single stranded DNA genome of ∼2800 nt to 3900 nt divided into two parts: a coding region (∼2150–2600 nt) and a non coding region (∼700–1200 nt). The coding region contains two ORFs, ORF1 and ORF2 encoding for the capsid and non structural proteins, respectively [32]. From our data set, we identified many reads showing a significant homology with circoviruses. We used a portion (∼90 amino acids residues) of the polymerase gene for phylogenetic analysis of our sequences and those downloaded from public domain databases. The phylogenetic tree generated using sequences of the replicase gene and shown in Figure 7 revealed an unexpected and previously unreported diversity of this new group of viruses.

## Discussion

We have conducted a metagenomics analysis with the primary focus on the examination of the community of RNA viruses in a fresh water lake ecosystem. In order to also assess the seasonal variations of the communities of RNA viruses, we performed two samplings in June and November to capture the baseline viral community during two seasons (summer and fall). We established a tangential flow filtration system capable of handling relatively small sample volumes (∼70 liters) as compared to large samples (∼200 liters) volumes processed in previous studies. To the tangential flow filtration system we associated 1) an additional size selection filtration through 0.22 µM column to enrich for viral particles and 2) nucleases (DNase and RNase) treatment for the removal of naked contaminating nucleic acids. Because our primary interest was to focus on the ecology and discovery of RNA viruses, enriched viral particles were processed for the preparation of total viral RNA. Given the expected complexity of the total viral RNA samples with the possibility that some viral types could be represented at very low levels, we needed to use a library construction method that includes an amplification step. Thus we used the RP-SISPA procedure [14] to construct a library of amplified DNA fragments from each total viral RNA sample. For each sample, the resulting library of DNA fragments ranging from 400 to 1000 nt was either used to construct a plasmid library by cloning into the topoTA vector and random recombinant plasmid clones sequenced using the Sanger method or sequenced directly using the 454 pyrosequencing method. Initially, using the Sanger method, we sequenced a random selection of approximately 4000 recombinant plasmid clones from each of the two plasmid libraries. After a preliminary data analysis that revealed great sample diversity, we proceeded to sequence many more DNA fragments using the 454 pyrosequencing. Overall, both 454 pyrosequencing and Sanger sequencing methods generated several thousands of sequence reads. Initial data analysis based on homology searches (BLASTX analysis) indicated the presence of a large proportion of viruses. However, as observed in other studies, a significant proportion of sequences reads could be identified as being related to known sequences of bacterial, archaeal and eukaryotic origins [33], [34]. Further analysis of the data especially in the context of the viruses detected demonstrated the presence of all four types of viruses including ssDNA, dsDNA, ssRNA and dsRNA viruses. The detection of all types of viruses, although in the cases of all DNA viruses represented unexpected contamination, underscored the sensibility and versatility of our method. Our ability to detect other types of viruses especially ssDNA viruses that would generally be difficult to detect unless special efforts are deployed to convert ssDNA into dsDNA prior to library construction and sequencing strengthens the utilization of the RP-SISPA for global (both RNA and DNA) viral metagenomics. The amplification-based library construction method used for this work also has other applications and hence can be utilized for the processing of samples for metagenomics analysis with limited amounts of nucleic acid materials. This may ultimately allow its use for multiple and small scales and frequent monitoring of a given environment or ecosystem for changes in the viral community over a period of time.

From the two data sets (November 2006 and June 2007), the majority of the sequences were unrelated to any previously published sequences but appeared to be most likely derived from novel viruses because known assemblies were 72–80% enriched for viral nucleic acids. This suggests that freshwater bodies as well as other marine environments are reservoirs for novel viruses [2], [35]. The presence of viral sequences with significant homology to insect, human and plant pathogens with relevance to human health and agriculture demonstrate another important outcome of investigating viral populations of freshwater bodies. Based on our methodology, we mostly expected the majority of the reads to be of RNA viral origin. The identification of DNA viruses and also sequences from other organisms (such as Archea, Eukaryotes and prokaryotes) may also contribute to identification of sequences of apparently unknown origin based on BLASTX analysis.

The identification of potentially novel viruses related to insect paralytic viruses has significance in light of a study involving the Israeli Acute Paralysis virus in bee Colony Collapse Disorder [31], [33]. The identification of another unexpected virus suggests that mosquitoes found around Lake Needwood may be infected with a member of the Seadornavirus genus with sequence similarity to the Banna virus. The similarity to Banna virus raises the interesting question of whether the Needwood Banna-like virus can infect humans as well. If yes, is it a potential agent of febrile illness in the USA as well? Until now, all members of Seadornaviridae have been found in Asia and it is unclear whether the Needwood Banna-like virus is endemic to North America or if it has recently been introduced into the USA.

In summary, our metagenomic study of two seasonal samplings of Lake Needwood has uncovered a tremendous diversity of RNA viruses in a freshwater body. The overall compositions of the data sets are similar to other metagenomic studies of RNA viruses in marine communities. This underscores great taxonomic diversity in both marine and freshwater communities. Additionally, we also demonstrated the presence of novel ssDNA viruses including a number of assemblies with some homology to ssDNA pathogenic viruses such as the porcine circovirus [31].

The great diversity of viruses present in the Lake may reflect the composition of organisms which live near or in Lake Needwood and also human activity in the area. A number of novel viral signatures were detected with possible implications on agriculture and public health. Further studies are required however, to investigate the ecology of these viruses and most importantly to determine their hosts. It should also be stressed, that while sequence comparison and phylogenetic analysis strongly suggest taxonomic relatedness between nucleic acid signatures detected in Lake Needwood and other known pathogenic viruses, nevertheless our data does not support the unequivocal identification of such novel viruses. With this caveat in mind however, this study demonstrates that data generated from metagenomic studies may serve as a preliminary baseline for monitoring viral complexity in the environment. Similar research could be extended to other freshwater bodies with geographic proximity to human residences (such as storm water detention ponds) for the establishment of a deep inventory of circulating viruses. Such additional studies would establish essential nucleic acid sequence baselines for the monitoring of disease-causing viruses that may have a significant impact on livestock, agriculture and human health as well as providing data with possible implications in the forecasting of emergence and re-emergence of viruses.

## Materials and Methods

### Study Site: Lake Needwood

Lake Needwood is a 75-acre man-made flood control lake in Montgomery County situated north of Rockville (Maryland, USA). The lake was constructed in 1965 to provide flood control draining an area of 12.8 square miles. In addition Lake Needwood was constructed to protect the water quality of the creek by functioning as a retention basin to trap all discharges from storm water runoff. It has a height of 65 feet with a length of 426 feet. The maximum discharge is 25652 cubic feet of water per second with a capacity of 7023 acre feet.

### Sample collection and purification of viral particles

We collected surface lake water and used a tandem tangential flow filtration system preceded by a series of 2.0 µM, 1.0 µM and 1 µM impact filters for the separation of cellular microorganisms from viral sized particles. The series of 2.0 µM, 1.0 µM and 1 µM filters was used to pre-clear the mixture. The first tangential filtration column (TFC) has a 0.22 µM cut off that allowed the viruses to go through but retained most bacteria. The second tangential filtration column unit has a 300,000 Daltons molecular weight cut off that retained viral sized particles. Using this system we concentrated ∼70 liters of pre-filtered lake water down to ∼120 ml from which viral particles were pelleted by ultracentrifugation at 149, 000 x g. The viral pellet was re-suspended with ∼2 mls of sterile PBS buffer.

### Enrichment of viral particles, library construction and DNA sequencing

Intact viral particles were first treated with nucleases (DNase I and RNase A) to remove contaminating nucleic acids from lysed cells that might have been in the concentrate. Viral RNA was then isolated using the Qiagen viral RNA preparation kit. Total viral RNA was subjected to random priming mediated sequence independent single primer amplification (RP-SISPA), a procedure that we have adapted for viral genome sequencing [14]. The RP-SISPA methodology consists of reverse transcription of viral RNA using random hexamers linked to a 20 nt known sequence. After the reverse transcription reaction, excess random hexamers anneal to the resulting cDNA followed by gap filling by Klenow DNA polymerase. The Klenow reaction product is used as template for PCR using a single primer representing the 20 nt of the chimeric reverse transcription oligonucleotide. Agarose gel analysis of the PCR reaction indicated amplicons with sizes ranging from 300 to 2600 nt. Amplicons of 500 to 1000 nt were gel purified. For Sanger sequencing, a small aliquot of size selected amplicons was ligated into a topoTA (Invitrogen) vector to generate a library of random DNA fragments. Random recombinant clones were selected from the TA vector library. For pyrosequencing, size selected PCR products were directly processed for adaptor ligation, library construction and emulsion PCR as recommended by the 454 pyrosequencing protocol.

### Data analysis

Sequences reads generated using the Sanger method and by the 454 pyrosequencing were combined and assembled using a hybrid assembly approach developed at the JCVI [18]. We processed both Sanger and 454 pyrosequencing sequences reads in a hybrid assembly. A hybrid assembly strategy significantly increased the number of contiguous (contigs) sequences with over 500 bp in size. BLASTX analyses were performed by searching the CAMERA non-redundant amino acid database – an extended version of NCBI's non redundant amino acid sequences database. We performed BLASTX and BLASTN (for ribosomal RNA sequences identification) searches. Individual reads and assembled sequences were assigned identities based on significant a BLASTX match with an e-value of 10e^−5^ or lower. These identities were then used for all subsequent taxonomical classification.

### Phylogenetic analysis

ClustalW was used to perform multiple sequence alignments of the sequences associated with selected reads and assembled reads into contigs. Previously described sequences of known viruses included in the phylogenetic analysis were downloaded from the NCBI and the GOS databases. As part of the phylogenetic analysis, all the alignments were performed with ClustalX 1.83 using the default pairwise alignment parameters (Gap Opening = 10.0, Gap extension = 0.1, Gonnet 250 protein weight matrix). Consensus tree bootstrapping was performed with Geneious 4.0.4 using the neighbor-joining method and 1,000 samples.

The following sequences with corresponding GenBank accessions numbers were used for respective phylogenetic analysis.

#### Banna viruses (vp1 sequences)

banna-1-vp1 china (AAF77631.1), banna-2-vp1 Vietnam (ACA50122.1), banna-3-vp1 Vietnam (ACA50110.1), banna-4-vp1 (NP_694469.1), banna-5-vp1 (AAF78849.1), kadipiro-vp1 (NP_694468.1), liao-ning-1-vp1 (YP_460026.1), liao-ning-2-vp1 (AAQ83562.1), rota-vp1 human rotavirus A (ABU87858.1).

#### Banna viruses (vp2 sequences)

banna-1-vp2 (AF134526_1 VP2), banna-2-vp2 (ACA50123.1), banna-3-vp2 (ACA50111.1), banna-4-vp2 (NP_694475.1), banna-5-vp2 (AAF78855.1), kadipiro-vp2 (AAF78850.1), liao-ning-1-vp2 (YP_460027.1), liao-ning-2-vp2 (AAQ83563.1), rota-vp2 (AAU43798.1).

#### Paralysis viruses (capsids sequences)

Cricket-paralysis-virus (AAF80999.1), Drosophila-C-virus (NP_044946.1), Israel-acute-paralysis-virus-of-bees (YP_001040003.1), Acute-bee-paralysis-virus (NP_066242.1), Kashmir-bee-virus (NP_851404.2), Rhopalosiphum-padi-virus (NP_046156.1), Aphid-lethal-paralysis-virus (NP_733846.1), Taura-Syndrome-virus (NP_149058.1).

#### Circoviruses (replicase gene sequences)

Porcine circovirus (YP_077191.1), Columbid circovirus (NP_059527.1), Duck circovirus (YP_271918.1), Gull circovirus (YP_803546.1), Finch circovirus (YP_803549.1), Starling circovirus (YP_610960.1), Raven circovirus (YP_764455.1), Muscovy duck circovirus (YP_164517.1), Canary circovirus (NP_573442.1), Mulard duck circovirus (YP_209621.1), Goose circovirus (NP_150368.1), Bovine circovirus (NP_048061.1), Swan circovirus-1 (ABU48445.1).

## References

[1] Edwards RA, Rohwer F (2005). Viral metagenomics.. Nat Rev Microbiol.

[2] Finkbeiner SR, Allred AF, Tarr PI, Klein EJ, Kirkwood CD (2008). Metagenomic analysis of human diarrhea: viral detection and discovery.. PLoS Pathog.

[3] Zhang T, Breitbart M, Lee WH, Run JQ, Wei CL (2006). RNA viral community in human feces: prevalence of plant pathogenic viruses.. PLoS Biol.

[4] Nakamura S, Yang CS, Sakon N, Ueda M, Tougan T (2009). Direct metagenomic detection of viral pathogens in nasal and fecal specimens using an unbiased high-throughput sequencing approach.. PLoS ONE.

[5] Schoenfeld T, Patterson M, Richardson PM, Wommack KE, Young M (2008). Assembly of viral metagenomes from yellowstone hot springs.. Appl Environ Microbiol.

[6] Culley AI, Lang AS, Suttle CA (2003). High diversity of unknown picorna-like viruses in the sea.. Nature.

[7] Culley AI, Lang AS, Suttle CA (2007). The complete genomes of three viruses assembled from shotgun libraries of marine RNA virus communities.. Virol J.

[8] Culley AI, Lang AS, Suttle CA (2006). Metagenomic analysis of coastal RNA virus communities.. Science.

[9] Breitbart M, Rohwer F (2005). Here a virus, there a virus, everywhere the same virus?. Trends Microbiol.

[10] Angly FE, Felts B, Breitbart M, Salamon P, Edwards RA (2006). The marine viromes of four oceanic regions.. PLoS Biol.

[11] Short CM, Suttle CA (2005). Nearly identical bacteriophage structural gene sequences are widely distributed in both marine and freshwater environments.. Appl Environ Microbiol.

[12] Breitbart M, Miyake JH, Rohwer F (2004). Global distribution of nearly identical phage-encoded DNA sequences.. FEMS Microbiol Lett.

[13] Watanabe RA, Fryer JL, Rohovec JS (1988). Molecular filtration for recovery of waterborne viruses of fish.. Appl Environ Microbiol.

[14] Djikeng A, Halpin R, Kuzmickas R, Depasse J, Feldblyum J (2008). Viral genome sequencing by random priming methods.. BMC Genomics.

[15] Margulies M, Egholm M, Altman WE, Attiya S, Bader JS (2005). Genome sequencing in microfabricated high-density picolitre reactors.. Nature.

[16] Andersson AF, Lindberg M, Jakobsson H, Backhed F, Nyren P (2008). Comparative analysis of human gut microbiota by barcoded pyrosequencing.. PLoS ONE.

[17] Huson DH, Auch AF, Qi J, Schuster SC (2007). MEGAN analysis of metagenomic data.. Genome Res.

[18] Goldberg SM, Johnson J, Busam D, Feldblyum T, Ferriera S (2006). A Sanger/pyrosequencing hybrid approach for the generation of high-quality draft assemblies of marine microbial genomes.. Proc Natl Acad Sci U S A.

[19] Seshadri R, Kravitz SA, Smarr L, Gilna P, Frazier M (2007). CAMERA: a community resource for metagenomics.. PLoS Biol.

[20] Williamson SJ, Rusch DB, Yooseph S, Halpern AL, Heidelberg KB (2008). The Sorcerer II Global Ocean Sampling Expedition: metagenomic characterization of viruses within aquatic microbial samples.. PLoS ONE.

[21] Rusch DB, Halpern AL, Sutton G, Heidelberg KB, Williamson S (2007). The Sorcerer II Global Ocean Sampling expedition: northwest Atlantic through eastern tropical Pacific.. PLoS Biol.

[22] Tyson GW, Chapman J, Hugenholtz P, Allen EE, Ram RJ (2004). Community structure and metabolism through reconstruction of microbial genomes from the environment.. Nature.

[23] Tringe SG, von MC, Kobayashi A, Salamov AA, Chen K (2005). Comparative metagenomics of microbial communities.. Science.

[24] Bench SR, Hanson TE, Williamson KE, Ghosh D, Radosovich M (2007). Metagenomic characterization of Chesapeake Bay virioplankton.. Appl Environ Microbiol.

[25] Garcia MH, Ivanova N, Kunin V, Warnecke F, Barry KW (2006). Metagenomic analysis of two enhanced biological phosphorus removal (EBPR) sludge communities.. Nat Biotechnol.

[26] Martin-Cuadrado AB, Lopez-Garcia P, Alba JC, Moreira D, Monticelli L (2007). Metagenomics of the deep Mediterranean, a warm bathypelagic habitat.. PLoS ONE.

[27] Nabeshima T, Thi NP, Guillermo P, Parquet MC, Yu F (2008). Isolation and molecular characterization of Banna virus from mosquitoes, Vietnam.. Emerg Infect Dis.

[28] Chen B, Tao S (1996). Arbovirus survey in China in recent ten years.. Chin Med J (Engl).

[29] de Miranda, Drebot M, Tyler S, Shen M, Cameron CE (2004). Complete nucleotide sequence of Kashmir bee virus and comparison with acute bee paralysis virus.. J Gen Virol.

[30] Van MM, Dullemans AM, Verbeek M, Van Den Heuvel JF, Clerivet A (2002). Sequence analysis and genomic organization of Aphid lethal paralysis virus: a new member of the family Dicistroviridae.. J Gen Virol.

[31] Allan GM, Ellis JA (2000). Porcine circoviruses: a review.. J Vet Diagn Invest.

[32] Biagini P (2004). Human circoviruses.. Vet Microbiol.

[33] Cox-Foster DL, Conlan S, Holmes EC, Palacios G, Evans JD (2007). A metagenomic survey of microbes in honey bee colony collapse disorder.. Science.

[34] Breitbart M, Hewson I, Felts B, Mahaffy JM, Nulton J (2003). Metagenomic analyses of an uncultured viral community from human feces.. J Bacteriol.

[35] Bench SR, Hanson TE, Williamson KE, Ghosh D, Radosovich M (2007). Metagenomic characterization of Chesapeake Bay virioplankton.. Appl Environ Microbiol.

